# BannMI deciphers potential *n*-to-1 information transduction in signaling pathways to unravel message of intrinsic apoptosis

**DOI:** 10.1093/bioadv/vbad175

**Published:** 2023-11-29

**Authors:** Bettina Schmidt, Christine Sers, Nadja Klein

**Affiliations:** Research Center Trustworthy Data Science and Security, Universitätsallianz Ruhr, 44227 Dortmund, North Rhine-Westphalia, Germany; Department of Computer Science, Humboldt-Universität zu Berlin, 10099 Berlin, Germany; Institute of Pathology, Charité–Universitätsmedizin Berlin, Freie Universität Berlin, Humboldt-Universität zu Berlin, and Berlin Institute of Health, 10117 Berlin, Germany; Department of Biology, Humboldt-Universität zu Berlin, 10099 Berlin, Germany; Research Center Trustworthy Data Science and Security, Universitätsallianz Ruhr, 44227 Dortmund, North Rhine-Westphalia, Germany; Department of Statistics, Technische Universität Dortmund, 44227 Dortmund, North Rhine-Westphalia, Germany

## Abstract

**Motivation:**

Cell fate decisions, such as apoptosis or proliferation, are communicated via signaling pathways. The pathways are heavily intertwined and often consist of sequential interaction of proteins (kinases). Information integration takes place on the protein level via *n*-to-1 interactions. A state-of-the-art procedure to quantify information flow (edges) between signaling proteins (nodes) is network inference. However, edge weight calculation typically refers to 1-to-1 interactions only and relies on mean protein phosphorylation levels instead of single cell distributions. Information theoretic measures such as the mutual information (MI) have the potential to overcome these shortcomings but are still rarely used.

**Results:**

This work proposes a Bayesian nearest neighbor-based MI estimator (BannMI) to quantify *n*-to-1 kinase dependency in signaling pathways. BannMI outperforms the state-of-the-art MI estimator on protein-like data in terms of mean squared error and Pearson correlation. Using BannMI, we analyze apoptotic signaling in phosphoproteomic cancerous and noncancerous breast cell line data. Our work provides evidence for cooperative signaling of several kinases in programmed cell death and identifies a potential key role of the mitogen-activated protein kinase p38.

**Availability and implementation:**

Source code and applications are available at: https://github.com/zuiop11/nn_info and can be downloaded via Pip as Python package: nn-info.

## 1 Introduction

Signal propagation in molecular networks can be abstracted to a set of interfaces of *n*-to-1 communication in which many senders concurrently “talk” to one receiver. Examples of such interfaces are the posttranslational modification of proteins or the orchestration of gene expression via signaling pathways. In more detail, this means that proteins, such as tumor suppressor p53 are phosphorylated at several residues simultaneously upon activation (Lavin and Gueven 2006, Liebl and Hofmann 2019). An *n*-to-1 example on the genetical level comes from the B-cell lymphoma 2 (Bcl-2) family, where the expression of various subsets of this family is governed by several signaling pathways. This can be fatal for a cell, as an imbalance of particular Bcl-2 members initiates the cell fate decision of apoptosis (Ramesh *et al.* 2009, Wang *et al.* 2022).

In this work, we provide evidence that this complex interplay cannot be investigated sufficiently by consideration of 1-to-1 signals only. However, predictions of state-of-the-art network inference methods such as STASNet (Dorel *et al.* 2018) or CellOracle (Kamimoto *et al.* 2023) are based on the analysis of 1-to-1 signals. In the following motivational example, we provide evidence how this can miss crucial interactions. To solve the issue for *n*-to-1 network interfaces in general, we present BannMI—a Bayesian nearest neighbor (NN)-based mutual information (MI) estimator. While BannMI can be applied on any data drawn from continuous variables, the focus of this work is its application on phosphoproteomic data.

### 1.1 Motivational example

Let $X=\{X_{1},X_{2},\ldots ,X_{n}\}$, $X_{i}=(X_{i,1},X_{i,2})$ be an *n*-sized sample of 2D uniform, independent and identically distributed (i.i.d.) random variables with no componentwise dependencies. Further, let $Z_{i}=\phi_{2}(X_{i})\;+\;Y_{i}$, where $\phi_{2}$ is the probability density function (pdf) of a standard 2D Gaussian distribution $N_{2}(0,\Sigma_{2})$, and $Y_{i}\;\overset{\mathrm{iid}}{∼}\;N(0,0.01)$, i=1,…,n. Intuitively speaking, we expect that $X_{i}$ is more informative for $Z_{i}$ if the covariance matrix $\Sigma_{2}$ of $\phi_{2}$ has componentwise correlations (plotted data of this example is shown in the Supplement). This intuition, which corresponds to a multivariate data dependency, is captured by the bivariate MI value, see Table 1 (last row). However, as the first two rows of the table illustrate, it is not possible to capture this effect via Pearson correlation or univariate MI. In this work, we take advantage of this new multivariate perspective to further understand potential information transduction in apoptosis.

**Table 1.** Quantifying multivariate data dependency.^a^

Measure	Uncorrelated	Correlated
Pearson correlation	−0.633	−0.167
Univariate MI	0.673	0.711
Bivariate MI	1.184	3.316

a Linear correlation between $X_{\cdot ,1},Z$ (first row), univariate/bivariate MI between $X_{\cdot ,1},Z$ (second row)/*X*, *Z* (third row), respectively. MI values are derived via BannMI.

Our case study on breast cancer data (Tognetti *et al.* 2021) provides evidence for such cooperative signaling in the apoptotic process. Further, via separate investigation of cell fate “phenotypes”, we identify a potentially fateful role of phosphorylated p38 in the apoptotic process. In addition, when comparing the potential, apoptotic signals of control cells and cancer cells, we find that the latter is significantly reduced. By that, we directly address Zielińska and Katanaev (2019), who suggest information theory to analyze signal alterations in cancer cells.

The undeniable asset of multivariate quantification of dependency has recently been demonstrated by the work of Erman (2023). Here, the author analyzed the molecular dynamics of a structural domain found in a broad variety of signaling proteins. To do so, MI was approximated with help of tensor Hermite polynomials. Here, it was found that in particular the dynamics of triplets of residues and not pairs of residues were altered in the presence of mutations. Uda *et al.* (2013) provides a rare example where *n*-to-1 communication is quantified in systems biology. To do so, empirical MI estimation based on descriptive histograms was used. More recently, Wada *et al.* (2021) concluded in their review that intracellular cell-to-cell heterogeneity, which is caused by the stochasticity of signal propagation, serves as information and not as noise in signaling. For their analysis, once again, the authors used mutual information. In the review Uda (2020), the author discusses diverse use-cases of MI in systems biology in theory. Finally, Karolak *et al.* (2021) discuss applications of information theory in systems biology with focus on cancer.

The paper is structured as follows: Section 2 (Methods) is a formal introduction of the information theoretical measures entropy, KLD, MI and channel capacity (CC). In this section we also provide a derivation of BannMI. In Section 3, we benchmark BannMI against state-of-the-art NN methods for MI on synthetic data with data generating processes that try to mimic the characteristics of phosphoproteomic data. In our case study we perform 1-to-1 MI/*n*-to-1 MI signal analysis for apoptosis on breast cell lines *per se* (Section 4.1). Finally, we conclude with a cell fate phenotypic signal analysis (Section 4.2).

## 2 Methods

BannMI estimates MI via the KLD. Both quantities are closely related to entropy, which forms the core of information theory. This familiarity explains why state-of-the-art estimators for entropy (Kozachenko and Leonenko 1987), MI (KSG; Kraskov *et al.* 2004), and KLD (Wang *et al.* 2009) apply the same pdf approximation. This approximation is rooted in Lebesgue’s differentiation theorem and applies NN distances. We describe Lebesgue’s approximation and derive the three NN distance-based estimators in the Supplementary Material. In contrast, our method approximates the ratio of two pdf’s and therefore is based on NN ratios.

In this section, we briefly introduce the information theoretical measures. Then, we review the KLD NN ratio estimator proposed by Noshad *et al.* (2017), which our BannMI extends to a Bayesian framework.

### 2.1 Preliminary

#### 2.1.1 Notation

Let *P* be a continuous probability measure on $\left(R^{d},B(R^{d})),\;d\in N\setminus \{0\right\}$ with probability density function (pdf) *p*, where $B(R^{d})$ is the Borel σ-Algebra on $R^{d}$. Let $x\in R^{d}$ and $n_{x}$ be an open neighborhood around *x*. Then, the support of *P* is defined as

$$S:=\{x\in R^{d}\;|\;\forall n_{x}\in B(R^{d}):\;P(n_{x})>0\}.$$

Let *Q* be a continuous probability measure on the same support with pdf *q*. Then, the Radon–Nikodym derivative of *p* with respect to *q* is

$$\tau (x):=\frac{p(x)}{q(x)}={\left(\frac{q(x)}{p(x)}\right)}^{-1},\;\forall x\in S.$$

Furthermore, the differential entropy of *P* is $H(p):=-\int_{S}\;\text{log}\;(p)p(x)dx,$ where *dx* is the Lebesgue measure on $\left(R^{d},B(R^{d})\right)$. Further, the KLD of *P* with respect to *Q* is

D(pq):=∫S log (p(x)q(x))p(x)dx=∫S log (τ(x))p(x)dx.

Now, let dimension d≥2 such that $P=P_{1}\times P_{2}$. Note that if d>2, at least one of the marginal distributions $P_{1},P_{2}$ is multidimensional. Further, let $p_{1}$ and $p_{2}$ be the respective pdfs. The MI of $P_{1}$ with respect to $P_{2}$ (and vice versa) is

(1) $$I(p_{1},p_{2}):=\int_{S}\;\text{log}\;\left(\frac{p(x)}{p_{1}(x_{1})p_{2}(x_{2})}\right)p(x)dx.$$

It is easy to see, that for $q(x)=p_{1}(x_{1})p_{2}(x_{2})$, the KLD is equivalent to MI. Further, channel capacity (CC) is defined as the maximum MI, given all feasible marginal probability measures $P_{2}$. That is

$$C(p_{1},p_{2}):=\underset{p_{2}}{\max}I(p_{1},p_{2}).$$

### 2.2 Estimation via NN ratios

#### 2.2.1 Notation

Let $X=\{X_{1},X_{2},\ldots ,X_{n}\}$ with n∈N be an i.i.d. sample with $X_{i}∼P$ and let ‖⋅‖ be a norm on $R^{d}⊃S$. Further, let $Y=\{Y_{1},Y_{2},\ldots ,Y_{n}\}$ be another i.i.d. sample with $Y_{i}∼Q$, with the same support as *P*. Then, $nn_{i,1}\in (X\cup Y)\setminus \left\{X_{i}\right\}$ is the first NN of $X_{i}$ in the joint sample if and only if

$$\left\|X_{i}-nn_{i,1}\right\|\leq \left\|X_{i}-Z_{j}\right\|\;\forall Z_{j}\in X\cup Y\setminus \{X_{i}\}.$$

For every positive integer k<2n, the *k*th NN $nn_{i,k}$ of $X_{i}$ is defined accordingly.

In the joint sample X∪Y, let $R_{i,k}=\{nn_{i,1},\ldots ,nn_{i,k}\}$ be the set of the *k* NNs of $X_{i}$. Further, define the number of NNs of $X_{i}$ in *X* and *Y* as $N_{i,k}=|R_{i,k}\cap X|$ and $M_{i,k}=k\;-\;N_{i,k}$, respectively. For 0<ϵ≤1, an *f*-divergence estimator is

(2) $$\begin{array}{c} {\hat{D}}_{n,k}^{N}(X,Y):=\max\left\{\frac{1}{n}\sum_{i}^{n}\tilde{f}\left(\frac{M_{i,k}}{N_{i,k}+1}\right),0\right\}\\ \text{with}\;\tilde{f}(x):=\max\left\{f(x),f\left(\epsilon \right)\right\}. \end{array}$$

If we insert f(x)=−log(x), an estimator for the inverse KLD is derived. If we allow $nn_{i,1}\in X\cup Y$, then the NN of $X_{i}$ is $X_{i}$ itself and the +1 in the denominator of [Equation (2)] becomes redundant. The interpretation of this NN ratio is as follows: Radon–Nikodyn derivative $\tau (X_{i})$ is considered as odds of a 0–1 random variable $B_{i}$, which is drawn from *P* rather than *Q* for a fixed $X_{i}$. That is

$$B_{i}∼{\text{Ber}}_{\theta_{i}}\;\text{with}\;\theta_{i}:=\frac{p(X_{i})}{p(X_{i})+q(X_{i})}.$$

Using $R_{i,k}$, the parameter $\theta_{i}$ can be estimated via its maximum likelihood estimator (MLE) given by ${\hat{\theta }}_{i,\text{MLE}}:=N_{i,k}/k.$ For *M_i,k_* ≠ 0, an estimator of the Radon–Nikodym derivative is thus

(3) $$\hat{\tau }(X_{i})=\frac{{\hat{\theta }}_{i,\text{MLE}}}{1-{\hat{\theta }}_{i,\text{MLE}}}=\frac{N_{i,k}}{M_{i,k}}.$$

We have shown that [Equation (2)] is based on MLE estimation. But the frequentist approach can be inferior to a Bayesian approach in cases of small sample size and biased data (Gelman *et al.* 2015). In the application considered, *k* defines $R_{i,k}$, which is the dataset used for MLE estimation for each i=1,…,n. Here, the choice of *k* is a tradeoff between sample size and biasedness of the data: For a small *k*, $\left|R_{i,k}\right|$ is small but the neighboring points $nn_{i,j}$ with j∈{1,2,…,k} are likely to be “close” to $X_{i}$. This implies $\frac{p(nn_{i,j})}{p(nn_{i,j})+q(nn_{i,j})}$ to be close to $\theta_{i}$, which is the parameter of interest. On the other hand, for a large *k*, sample size is increased at the cost of biased data. Those drawbacks motivated our Bayesian approach.

### 2.3 A Bayesian NN-based KLD estimator

As shown in [Equation (3)], the issue of KLD estimation can be reduced to estimation of a success-parameter $\theta_{i}$, for every data point $X_{i}$ in the sample *X*. In a Bayesian framework, estimation of $\theta_{i}$ is a statistic of the Beta-binomial distribution. We choose a natural, conjugate prior $\theta_{i}∼\text{Beta}(\alpha ,\beta )$. In the following, we refer to its parameters as $\hat{\alpha }$ and $\hat{\beta }$. This leads to a NN-based Bayesian KLD estimator

(4) $${\hat{D}}_{n,k}^{B}(X,Y):=\frac{1}{n}\sum_{i=1}^{n}\;\text{log}\;\left(\frac{N_{i,k}+\hat{\alpha }}{M_{i,k}+\hat{\beta }}\right).$$

The posterior distribution for each $\theta_{i}$ is again Beta-distributed with parameters $\alpha_{i}=\hat{\alpha }+N_{i,k}$ and $\beta_{i}=\hat{\beta }+M_{i,k}$. By that, a posterior mean estimate of $\theta_{i}$ is

(5) $${\hat{\theta }}_{i,B}=\frac{\alpha_{i}}{\alpha_{i}+\beta_{i}}=\frac{\hat{\alpha }+N_{i,k}}{\hat{\alpha }+\hat{\beta }+k}.$$

Now, plugging ${\hat{\theta }}_{i,B}$ into the formula leads to

$${\hat{\tau }}_{B}(X_{i})=\frac{{\hat{\theta }}_{i,B}}{1-{\hat{\theta }}_{i,B}}=\frac{N_{i,k}+\hat{\alpha }}{M_{i,k}+\hat{\beta }}.$$

Remark 1 In Section 3, we test an empirically derived set of $\left\{\hat{\alpha },\hat{\beta }\right\}$ using the method of moments as proposed by Gelman et al. (2015). See the Supplementary Material for further details.

### 2.4 BannMI—a Bayesian NN-based MI estimator

As derived in Section 2.1, the MI is an application of KLD, when $Q=P_{1}\times P_{2}$ and $q(x_{1},x_{2})=p_{1}(x_{1})p_{2}(x_{2})$. Accordingly, one can estimate the MI between the lower dimensional subsets $X^{1}=\{X_{1,1},\ldots ,X_{n,1}\}$ and $X^{2}=\{X_{1,2},\ldots ,X_{n,2}\}$ of X via a random shuffling of elements in $X^{2}$ such that ${\tilde{X}}^{2}=\{X_{(1),2},X_{(2),2},\ldots ,X_{(n),2}\}$ is independent from $X^{1}$. Our BannMI is then defined as

(6) $${\hat{I}}_{n,k}^{B}(X^{1},X^{2}):={\hat{D}}_{n,k}^{B}((X^{1},X^{2}),(X^{1},{\tilde{X}}^{2})).$$

By making use of this equivalence between KLD and MI, our BannMI in [Equation (6)] allows us to quantify information propagation in molecular networks. Please see the Supplement for its detailed application in this context.

## 3 Benchmark study

In this section, we conduct three studies to benchmark the performance of BannMI against selected competitors. These are the state-of-the-art NN MI estimator KSG (Kraskov *et al.* 2004) and the KLD estimator based on NN distances (Wang *et al.* 2009); see the Supplementary Material for details on both approaches. As in Equation (1), we apply the latter as estimator for the MI and refer to it as WMI. Furthermore, we use the frequentist NN ratio-based KLD estimator as presented in Equation (2) and apply it for MI estimation (NMI). Here, we may note that to the best of our knowledge both KLD estimators have not been applied as MI estimators so far. In particular, the utilization of Equation (2) as MI estimator requires several steps, such as the choice of the *f*-function, the choice of a suited ϵ parameter (see Supplementary Material) as well as the approach of data shuffling (see Section 2.4). The reader finds our collection of new MI estimators as well as the established KLD estimators implemented in our Python package nn-info.

Due to our precise application goal of MI estimation on phosphoproteomic data, the aim of this paper is not to construct an estimator that performs well on any feasible pair of distributions, in any dimensionality and preferably on any sample size *n*. Rather, in this benchmark we focus on good (qualitative) performance on simulated data that shares the characteristics of phosphoproteomic data. Some of those characteristics are a sample size with a magnitude of $10^{4}$, nonnegativity and skewness of the data and a high componentwise dependency.

Optimal parameter settings of the implemented algorithms were either derived in the KLD benchmark that can be found in the Supplementary Material. Or/and, the parameters were further tested for optimality in this study. Here, we point out the importance of the nearest neighbor parameter *k*. As NMI/BannMI are both nearest neighbor ratio estimators on the one hand, and WMI/KSG are both nearest neighbor distance estimators on the other hand, the optimal choice of *k* varies greatly. As in the KLD benchmark, performance of BannMI with empirically derived hyperparameters was comparable to a setting where $\hat{\alpha }=\hat{\beta }=0.1$, we include both approaches in the following procedures and refer to the latter as “uBannMI”. The following benchmark study was conducted on three data generating processes (DGP)s.

### 3.1 Multivariate Gaussian distribution

DGP: First, we derived 62 covariance matrices from phosphoproteomic data presented by Tognetti *et al.* (2021) for d∈{2,5,10} (all cell lines, condition: EGF stimulation, time point t=0). Next, we sampled centered Gaussian data from those covariance matrices $\Sigma \in R^{d\times d}$. We tested performances for two different choices of *k* which produced best results in the KLD benchmark procedure (see Supplementary Material).

**Figure 1.**
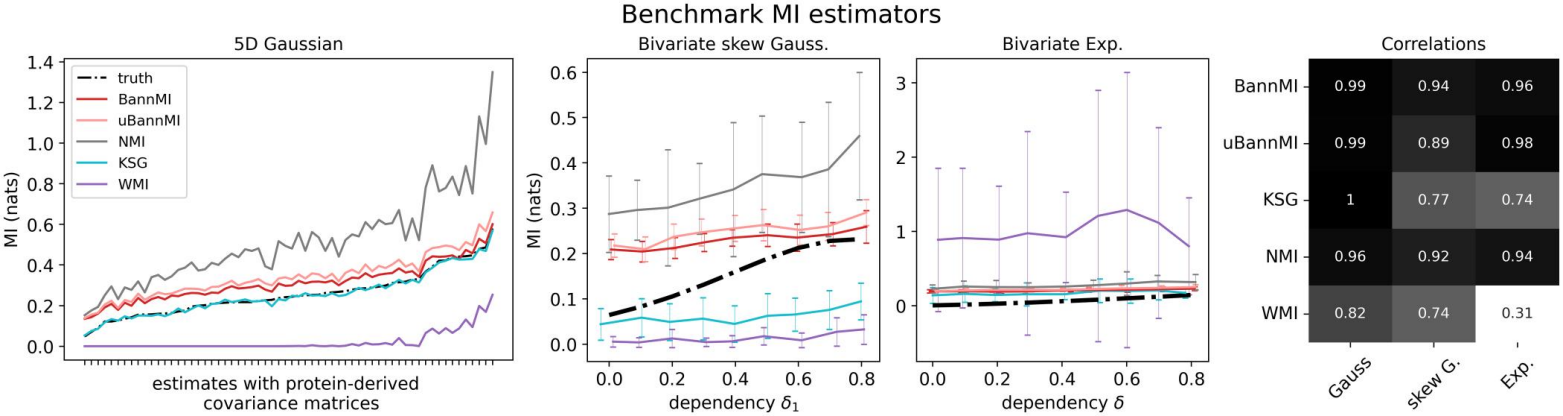
Left: Performance of MI estimators on 5D Gaussian data sampled from 62 protein-like covariance matrices (*x*-axis), shown together with the respective analytical MI values. Each estimate is a mean of 25 computations with sample size *n* = 1000. Center: Performance of MI estimators on bivariate skew Gaussian data (left) and bivariate exponential data. *X*-axis show values for increasing componentwise dependency, as indicated by dependency parameters $\delta_{1}$ and δ. Depicted are means and standard deviations of 25 computations with sample size *n*=1000 each. Approximations of the true values were derived by numerical integration via nquad in Python. Right: Pearson correlation between MI estimates and analytical value (Gaussian)/numerical estimates (others).

Main results: We find that in the Gaussian application, the empirical version of BannMI performs second best after KSG with respect to MSE/standard deviation and Pearson correlation. See extensive results in the Supplementary Material. Figure 1 (left) depicts an performance example of the estimators for d=5 and k=10 (BannMI, uBannMI, NMI), k=1 (KSG) and k=2 (WMI).

### 3.2 Multivariate skew Gaussian distribution

DGP: Phosphoproteomic data are likely to be skewed and on $R^{+}$. To take this setting into account, skew Gaussian data are simulated for 2D as described in Azzalini and Valle (1996). We apply the dependency parameter $\delta ={\left(\delta_{1},\delta_{2}\right)}^{⊤}$ as follows: While $\delta_{2}=0.5$ remains fixed, we perform experiments for $\delta_{1}\in \{0.0,0.1,\ldots ,0.8\}$. Then, we sample $Y_{0}$ from a 1D standard Gaussian distribution and $Y={\left(Y_{1},Y_{2}\right)}^{⊤}$ from a 2D Gaussian distribution with 0-mean vector and covariance matrix $\Sigma_{2}$. As in Section 3.1, $\Sigma_{2}$ is derived from a cell line phosphoprotein expressions. For j∈{1,2}, we finally compute the skew Gaussian random variables $X_{j}=\delta_{j}|Y_{0}|+{\left(1-\delta_{j}^{2}\right)}^{\frac{1}{2}}Y_{j}$ with dispersion matrix $\Sigma_{s}$ and skewness parameter *α*.

Main results: Figure 1 (center) shows results for increasing $\delta_{1}$ (*x*-axis). The plot shows mean values and standard deviations for all estimators. Here, Pearson correlation to the numerically estimated MI is highest for BannMI/NMI (see Fig. 1, right). However due to its lower standard deviation, the choice of BannMI is favorable.

### 3.3 Bivariate exponential distributions

**Figure 2.**
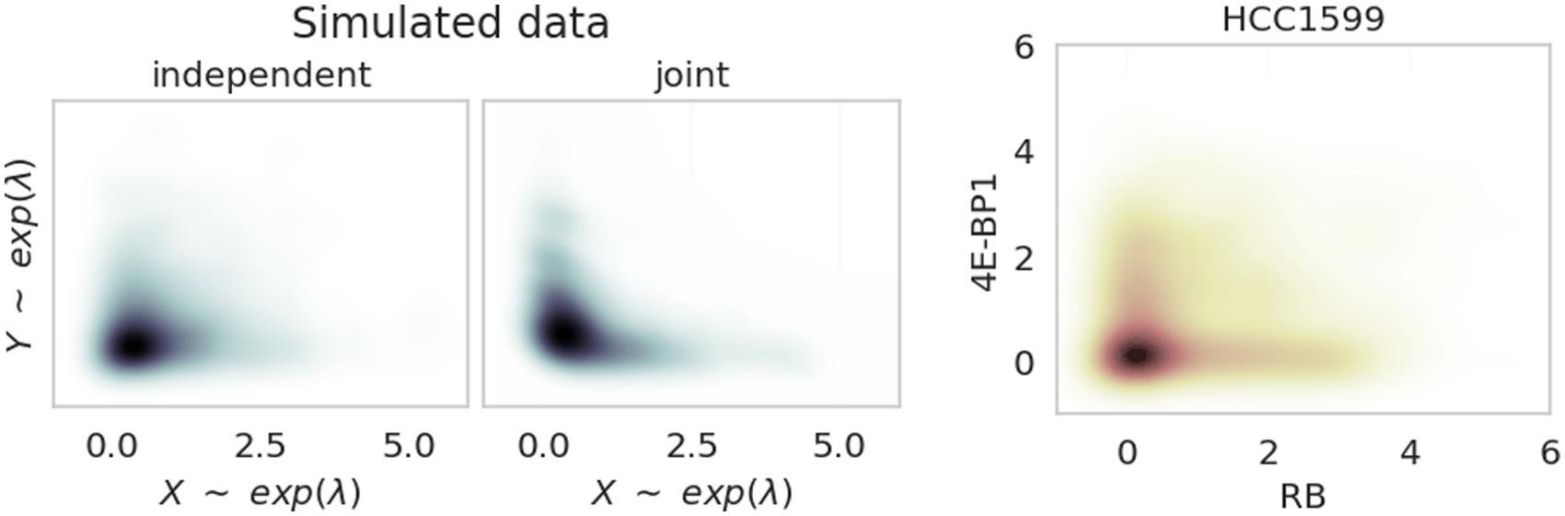
Density plots of simulated exponentially distributed data (left) and 2D phosphoprotein expression values (right) of breast cancer cell line HCC1599. The phosphoprotein expressions selected are the eukaryotic translation initiation factor 4E-binding protein 1 (4E-BP1) and the retinoblastoma protein (RB). Each plot is based on 1000 data points. In the simulated, dependent scenario, Gumbel’s method was used as described in Section 3.3.

DGP: So far, we tested our MI estimators for component-wise correlated symmetric and skewed data. Now, we further approximate the characteristics of phosphoproteomic data by consideration of nonlinear correlated data. Figure 2 (right) shows expressions of phosphoproteins RB and 4E-BP1 of the Tognetti *et al.* (2021) dataset. Both phosphoproteins represent an “either-or” scenario (XOR), which resembles the joint expression of two exponential variables, as shown next to it.

Gumbel (1960) suggested a dependency parameter δ∈(0,1) for two exponential variables, such that the joint cumulative distribution function is $F_{\delta }(x,y)=1-e^{-x}-e^{-y}+e^{-x-y-\delta xy}$. To simulate data, we used the No-U-Turn-Sampler (NUTS; Hoffman and Gelman 2014) from the PyMC3 package. We sampled bivariate random variables from $F_{\delta }$ with exponential priors for δ∈{0.0,0.1,…,0.8}. An approximation of the true MI value is derived via numerical integration.

Main results: Figure 1 (center) shows that the two NN distance-based MI estimators, KSG and particularly WMI, perform poorly for increasing dependency parameter δ (*x*-axis). With respect to standard deviations and the high Pearson correlation toward the numerically derived estimate, BannMI once again performs superior to NMI (Fig. 1, right).

## 4 Case study on CyTOF data

Motivated by our biological reasoning, we have so far postulated *n*-to-1 signaling and suggested MI for its analysis in single cells. This led to our Bayesian MI estimator based on NN ratios which is well suited for MI estimation on phosphoproteomic data. Next, we apply the algorithm on suitable *in vitro* data. Suitable in this sense means that firstly phosphoproteins measured should provide information about the activation/deactivation of signaling pathways; and secondly substrate expressions relevant for cell fate decisions should be available. In their 2021 paper, Tognetti *et al.* (2021) claim to have generated the largest multiplex single-cell signaling dataset to date. It consists of 67 human breast cell lines, among which 62 are cancerous. 37 Phosphoproteomic marker expression define the dimensionality of the data, among which are prominent signaling pathway members as well as markers for cell fate decisions such as apoptosis and cell cycle progression. Because of its sheer magnitude, we chose this dataset for our analytic proposes. This is the technical reason. A further reason for our choice is that despite current advances in precision medicine, some cases of breast cancer are still not responsive to treatment.

The dataset is a phosphoproteomic perturbation study. Among the cell lines are all relevant breast cancer subtypes, which are luminal/hormone receptor positive (HR+), HER2/ERBB2 positive (ERBB2+) and basal/triple negative breast cancer (TNBC). While the subtype classification into luminal/basal refers to the breast cell type that gave rise to the malignancy, the classification into HR+/TNBC refers to hormonal signaling. However, in most of the cases both classification schemes are interchangeable. HR+/TNBC cell lines are further classified into an A or B type, according to the PAM50 (Prediction Analysis of Microarray 50) gene set classification, or rather the gene cluster classification as proposed by Neve *et al.* (2006). Five noncancerous breast cell lines serve as control for nonpathological signaling.

As customary for signal pathway analysis, cells were stimulated with epidermal growth factor (EGF) after a period of cell growth (48–72 h) and a night of starvation. Data provided is a time series starting from zero minutes up to one hour after EGF stimulation. While EGF is a prominent growth factor in breast cancer, it has been shown before, that a subset of breast cancer cells also respond to EGF with cell cycle arrest and apoptosis (Ali *et al.* 2018). With help of the time series provided, data allows a sophisticated analysis of the MAPK signal progression, as demonstrated in Tognetti *et al.* (2021), with respect to kinase expression levels. It has been shown in Uda *et al.* (2013) that information propagation seems to be more robust than phosphoprotein expression levels. Early applications of BannMI on the time series data share this observation (data not shown).

Among the signaling phosphoproteins, several kinases have been identified/suggested as influential for intrinsic apoptosis. In the following analysis we refer to them as “effectors for apoptosis” (EfA)s; see Table 2 for their further characterization. Throughout the paper, EfA expressions refer to their phosphorylated state. For more information about the respective phosphorylation site measured, see Tognetti *et al.* (2021). Furthermore, the dataset contains expression of cleaved Caspase-3, a molecule that is cleaved in the ongoing process of apoptosis, which we will use as marker for apoptotic cells.

**Table 2.** Phosphoproteins and their potential role in the apoptotic process.^a^

Kinase	Effect on apoptosis	Selective reference
ERK1/2: Extracellular signal-regulated kinase 1/2	p53 Ser15 kinase	Yue and López (2020)
JNK: C-Jun N-terminal Kinase	p53 Thr81 kinase	Lee and Gu (2010)
p38: Mitogen-activated protein kinase p38	p53 Ser33 (and Ser46) kinase	Yogosawa and Yoshida (2018)
AMPK: AMP-activated protein kinase	p53 Ser46 kinase	Green *et al.* (2014)
STAT1: Signal transducer and activator of transcription 1	Cross-talk with p53	Zhang and Liu (2017)
STAT3: Signal transducer and activator of transcription 3	Interaction with Bcl-2	Grivennikov and Karin (2010)
Smad2/3: Mothers against decapentaplegic homolog 2 and 3	TGF-β-induced apoptosis (via Bim)	Ramesh *et al.* (2009)
GSK3β: Glycogen synthase kinase 3 beta	Promotion of DNA repair	Lin *et al.* (2020)
NFκB: Nuclear factor kappa B	Interaction with Bcl-2	Pires *et al.* (2018)
RB: Retinoblastoma protein	Interaction with Bcl-2	Polager and Ginsberg (2009)

a JNK as well as p38 have been suggested to play a role in p53 stabilization via p53 phosphorylation at Ser15 and Ser20 (Wu 2004). Other effectors for apoptosis used for analysis in Section 4 are the STAT family member protein STAT5 (Halim *et al.* 2020) and further the ratios STAT1/STAT3 as suggested by Avalle *et al.* (2012) and STAT1/STAT5. Further included is the Sarcoma proto-oncogene (Src).

We proceed as follows: First, we compare potential 1-to-1 signaling for apoptosis with the respective, potential 4-to-1 signaling in cancer cells and the control (Section 4.1). In Section 4.2, we further zoom into the data and investigate the potential signal per cell phenotype (apoptotic, proliferating or resting cells). Throughout this study, we focus on 4-to-1 signaling results that could not have been identified with a 1-to-1 analysis. In the Discussion, we provide a biological interpretation of our findings.

### 4.1 MI and channel capacity in programmed cell death

**Figure 3.**
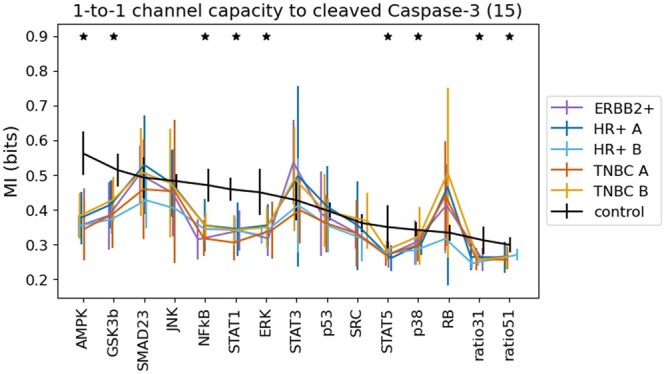
CC estimates between each phos. EfA (*x*-axis) and cleaved Caspase-3. Per cell line (downsampled, *n*=1000 each) a CC mean was derived from 15 computations. Then, results were grouped into control or cancer subtype. Standard deviations show variation among the groups. Stars indicate significant differences between cancer and control.

**Figure 4.**
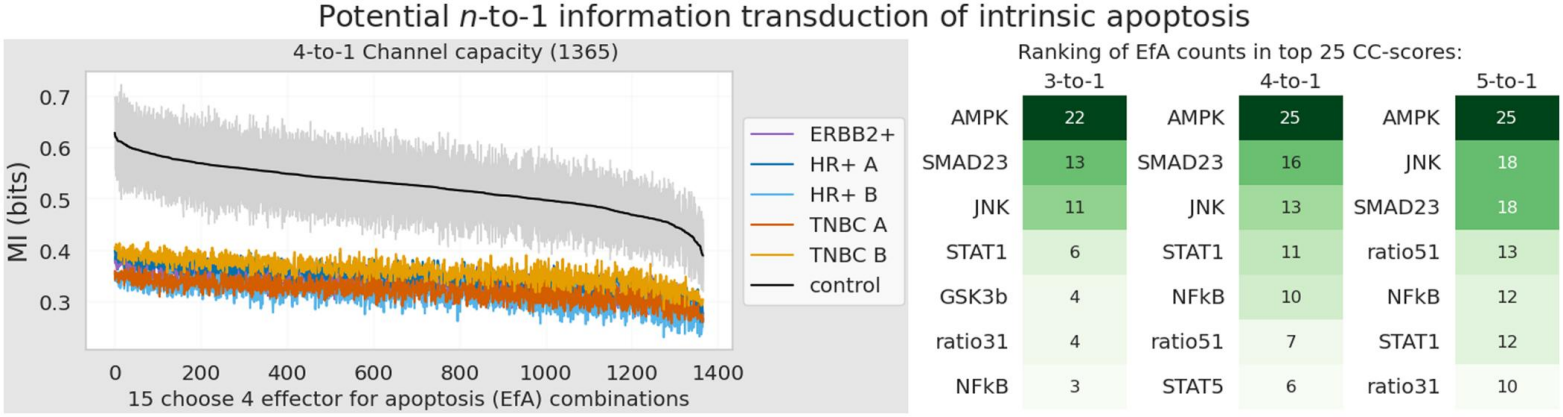
Left: mean CC (15 computations per cell line) for all 4D EfA combinations with respect to the apoptotic marker cleaved Caspase-3. Results were grouped into control and cancer subtype, then sorted with respect to decreasing CC value of the control cell lines. Shaded area marks standard deviations for the control cell line results. Right: EfA-quadruples of the 25 highest 4D CC scores were selected. Next, occurrences of the single EfAs in the 25 quadruples were ranked according to frequency (4-to-1). Analysis was repeated for a 3D and 5D signal.

Since the dataset provides only one dose of EGF stimulation and discrete time points, we approximated CC by choosing the time point t=40, as it maximized MI for most cell line settings. We first computed 1D CC toward cleaved Caspase-3 for the 15 EfAs as described in Table 2 (see Fig. 3). Significant CC differences (one-sided t-test: Welch, all cancer lines versus control) are marked with stars. To systematically unravel the dependency between EfAs and the apoptotic marker, we next applied the same approach of channel capacity to all $\left(\begin{array}{c} 15\\ 4 \end{array}\right)=1365$ 4D combinations of EfAs [see Fig. 4 (left)]. We observe that the tightly orchestrated structure, in which intrinsic apoptosis unfolds in healthy cells, is absent in cancer. Significant differences in channel capacity for control compared to cancer hold for all combinations (maximum p-value: 0.02).

To extract the impact of a single EfA within the 4D setting, we ranked all combinations with respect to CC, then selected the 25 top performing quadruples and count occurrences of each EfA [see Fig. 4 (right)]. It is of interest that the top reoccurring EfAs of that ranking procedure are not identical with the EfAs of the highest 1D CC scores. In particular, glycogen synthase kinase-3 β (GSK3β), which is associated with the DNA damage response (DRR) of a cell, is ranked second in the 1D scenario. However, the kinase is ranked only eighths in the 4D scenario with only 4 out of 25 occurrences. This might indicate that its signal is not cooperative in the considered framework. In contrast, in our analysis AMPK, Smad2/3 as well as JNK are tightly linked within the signal of apoptosis and therefore might act with “joint forces”. We repeated the analysis for 3D and 5D EfA combinations and received stable results with respect to significant control-cancer differences and EfA counts.

We further investigated the combinatorial effect of EfAs on apoptosis by training 4D random forests. In doing so, we found EfA combinations that are sufficient but not necessary to predict apoptosis in the control cells, see the Supplementary Material.

### 4.2 MI key players for apoptosis in apoptotic, proliferating, and resting cells

**Figure 5.**
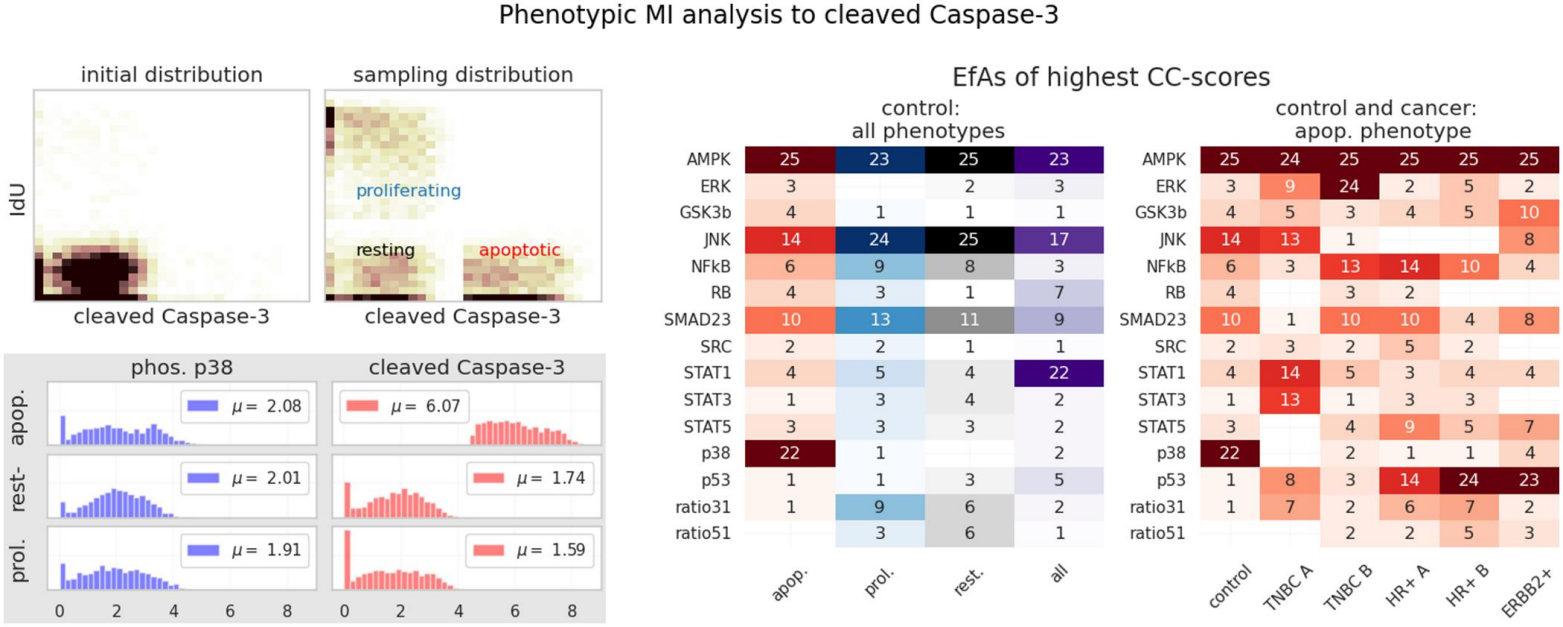
Image top left: Original phenotypic distribution in control cell lines with respect to both threshold markers IdU and cleaved Caspase-3; the bordering image shows the phenotypic distribution after the downsampling procedure. Center: EfA occurrence rankings with respect to CC per phenotype for the control cell lines. Right: EfA occurrence rankings for the control cell lines and the five breast cancer subtypes with respect to the apoptotic phenotype. CC values applied for the rankings were means of 15 computations with BannMI. Sample size for all phenotype experiments was *n*=1000. Bottom left: the phosphoprotein expression of p38 and cleaved Caspase-3 for all phenotypes (control). While cleaved Caspase-3 expression is strongly amplified in apoptotic cells (3-fold increase), p38 expression remains almost unchanged, as can be seen in the mean expression values μ.

So far, we used MI to identify an overall significantly lowered potential apoptotic signal in cancer cells when compared to noncancerous ones. However, apoptotic, as well as proliferating cells form a minority in the control cell data (5 cell lines). To zoom into the structural dependencies of those phenotypes, we subsampled this control data for phenotypes by thresholds: cells with 5-Iodo-2’-deoxyuridine (IdU) expression (a marker for S-phase in the cell cycle) above 3.5 are selected as proliferating, cells with cleaved Caspase-3 expression above 4.5 are selected as apoptotic, and cells that do not meet any of both criteria are defined as resting [see Fig. 5 (top left)]. We added a mixed sample of the three phentotypes and refer to it as “all”.

Analogue to Section 4.1, we computed 4D CC with respect to cleaved Caspase-3 and ranked EfA occurrences of the 25 highest CC-scores. Figure 5 (center) displays the computational results. Here, we want to raise attention to the ranking similarities of the proliferating and the resting cell phenotype: as in the previous subsection, a robust dependency of the identified potential apoptotic keyplayers AMPK, JNK and partially the Smad2/3 proteins Smad2 and Smad3 can be observed. Interestingly, this pattern reoccurs in the apoptotic cells, but in addition a robust dependency on MAPK pathway member p38 is observable.

We underline that this role of p38 could not have been found via a method based on differential mean value expression, as phosphorylated p38 is almost identically expressed in all cellular phenotypes, as can be seen in Fig. 5 (bottom left). Further, the Pearson correlation between phos. p38 and cleaved Caspase-3 is lowest in apoptotic cells (0.26), followed by 0.39 (proliferating) and 0.42 (resting).

So far, we restricted our phenotypic analysis to control cells to firstly understand “healthy” dependencies, as they might pave the way to understand causative apoptotic signaling. Next, we investigated the potential signaling roles of our EfAs in cancerous, apoptotic cells. We find that the consistent joint signal of phosphorylated AMPK, JNK, Smad2/3 with the switching role of p38 is absent in cancer cells [see Fig. 5 (right)]. Instead, the heterogeneity of cancer is demonstrated once again. Furthermore, these results indicate the clinical challenge to reconstruct an apoptotic signal in cancer cells. We hope that a joint signal analysis can help in this process of reconstruction.

Finally, we used BannMI as KLD estimator to investigate multivariate differential expression (DE) of the phosphorylated EfAs. We ranked EfA occurrences of 4D DE between the phenotypes in the control cells, as well as between control and cancer cells. See the results in the Supplementary Material.

## 5 Discussion

### 5.1 Biological interpretation

P53 is the central figure of intrinsic apoptosis. The transcription factor harbors a large number of phosphorylation sites that alter its functionality. According to Lavin and Gueven (2006), there are 11 serine activation sites (Ser6, Ser9, Ser15, Ser20, Ser33, Ser37, Ser46, Ser366, Ser376, Ser378, Ser392) and four further threonine activation sites (Thr18, Thr81, Thr377 and Thr387). Phosphorylation at Ser15, which was measured in the dataset by Tognetti *et al.* (2021), is mediated via the ataxia telangiectasia mutated (ATM) kinase, a central kinase regulating DNA damage response, and is essential for the transcriptional activation of p53 as follows. P53 is a short-lived protein that is degraded via ubiquitination via its main inhibitor, the mouse double minute 2 (Mdm2) protein in unstressed cells, such that only low expression levels occur in healthy cells. Phosphorylation of Ser15 (together with Ser20 and Thr81; Buschmann *et al.*, 2001) interrupts p53-Mdm2 interaction such that p53 accumulates and translocates into the nucleus. Therefore, phosphorylation of p53 at Ser15 is an indication for p53 activation and potential apoptosis induction in cancer cells. Other important p53 kinases are AMPK and JNK. Both phosphoproteins, together with the main TGF-β effectors Smad2/3 are the top occurring EfAs in the 4D CC analysis of Sections 4.1 and 4.2. In the latter, also phosphorylated p38 is among the top occurring EfAs, but only in the apoptotic phenotype. Therefore, our results indicate firstly that p53 phosphorylation is cooperative via AMPK, JNK and p38.

Secondly, the strong cooperation of Smad2/3 in the joint signal might provide further evidence for a control mechanism of apoptosis which is guarded by the signal of several pathways. Smad2/3 are the main kinases of the TGF−β pathway. The pathway is, among others, linked to apoptosis. Cordenonsi *et al.* (2007) show that phosphorylated p53 (at Ser6 and Ser9) physically interact with Smad2/3 and thus jointly regulates the transcription of several TGF-β target genes. Among those substrates is the Bcl-2-interacting mediator of cell death protein (Bim), a pro-apoptotic member of the Bcl-2 family. Its major isoform is BimEL (where the “EL” stands for “extra long”). Interestingly, it has been shown that BimEL ubiquitination and degradation is controlled by Erk1/2 phosphorylation (Ramesh *et al.* 2009). Those are only two examples of cooperative signaling between the MAPK and the TGF-β pathway. Recently, Wang *et al.* (2022) discuss a dual control model for intrinsic apoptosis and provide an alternative model based on *in vitro* experiments with cell lines of three cancer types. In those models, apoptosis “happens” in the onset of Bcl-2 protein interaction chains with a special focus on Bcl-2 homology 3 (BH3)-only proteins, such as Bim. The authors show with help of p53 deficient cells that Bim expression is independent of p53 in their experiments. However, apoptosis is mainly observed in p53 wild type cell lines.

### 5.2 Future applications of BannMI

We propose BannMI, an important tool to identify cooperative signaling and to disentangle individual roles in *n*-to-1 signaling based on NN MI estimation. Results of our case study indicate that some phosphoproteomic dependencies are only observable if more than one protein expressions/or their phosphorylations, are considered at once. This new perspective allows quantification of the complex interplay of several signaling pathways in cell fate decisions along the example of apoptosis in breast cell lines/breast cancer cell lines. It is of interest for future research if our findings can be extended to other cellular types besides the human breast.

The generality of BannMI allows to zoom into any scenario of *n*-to-1 communication with moderate dimensionality (benchmarking covered dimensionality up to d=10), which could be any kind of omics data.

## Supplementary Material

vbad175_Supplementary_Data

## Data Availability

Data are available on Synapse and Mendeley Data. For the latter, check https://data.mendeley.com/datasets/gvh2vtg86r/1.
