# Supplementary material for: BannMI deciphers potential n-to-1 information transduction in signaling pathways to unravel message of intrinsic apoptosis
Source: Bioinform Adv. 2023 Nov 29;4(1):vbad175. doi: 10.1093/bioadv/vbad175 (PMC10769817; doi:10.1093/bioadv/vbad175)
Supplement: vbad175_Supplementary_Data [file vbad175_supplementary_data.pdf]

1. Supplementary figure on the motivational example

As stated in the introduction, let $X = \{X_1, X_2, \ldots, X_n\}$, $X_i = (X_{i,1}, X_{i,2})$ be an n-sized sample of 2D uniform, independent and identically distributed (iid) random variables with no componentwise dependencies. Further, let $Z_i = \phi_2(X_i) + Y_i$, where ϕ_2 is the probability density function (pdf) of a standard 2D Gaussian distribution $\mathcal{N}_2(0, \Sigma_2)$, and $Y_i \overset{iid}{\sim} \mathcal{N}(0, 0.01)$, $i = 1, \ldots, n$. Figure 1 displays data examples of two different covariance matrices. Here, the example on the right hand-side has a higher covariance, which leads to a higher value for 2D MI estimation between X and Z.

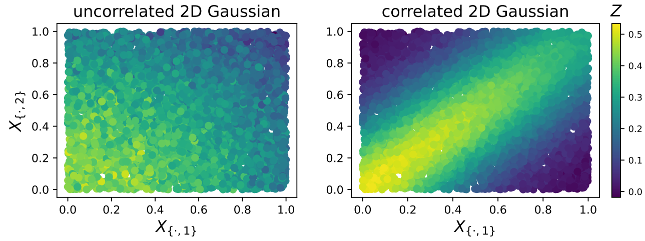

Fig. 1. $n = 5,000$ data points X_i, $i = 1, \ldots, n$. Left: $X_i = (X_{i,1}, X_{i,2}) \sim \mathrm{Unif}(0,1)^2$ and $Z_i = \phi_2(X_i) + \mathcal{N}_2(0, 0.01)$, where ϕ_2 is the pdf of a standard 2D Gaussian distribution with zero mean and unit covariance $\Sigma = I_2$. Right: $\Sigma_{1,2} = \Sigma_{2,1} = 0.95$.

2. Supplementary information: BannMI to quantify information propagation in molecular networks

Let $X = \{X_i\}_{i=1}^n$ with $X_i = (X_{i,1}, X_{i,2}, \ldots, X_{i,d})$ be a single cell sample with d measured phosphoproteins per single cell, such that $X_{i,s}$ with $s \in \{1, 2, \ldots, d\}$ is the expression/phosphorylation of protein s in single cell i and $X^s := \{X_{i,s}\}_{i=1}^n$ is the 1D sample of all n single cells. Now, suppose vector components $u = (u_1, u_2, u_3)$ with $u_j \in \{1, 2, \ldots, d\}$ correspond to upstream kinases of s (see Figure 2), such that $X_{i,u} = (X_{i,u_1}, X_{i,u_2}, X_{i,u_3})$ and $X^u := \{X_{i,u}\}_{i=1}^n$ is the 3D subsample of X consisting of the upstream kinases. Then, information propagation in this small molecular network can be measured by $\hat{I}^B_{n,k}(X^s, X^u)$, which is our BannMI. It quantifies the dependency between X^s and X^u. It is easy to see that this approach can be extended to molecular networks consisting of more than one interface.

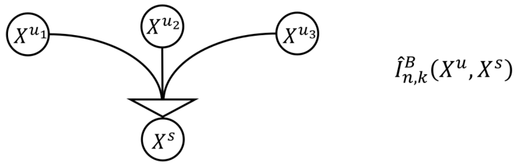

Fig. 2. Graphical intuition for n-to-1 communication measured with help of BannMI as opposed to a standard graph-based 1-to-1 communication

3. Supplementary material on methods

As mentioned in the Methods part of our paper, a popular approach to derive estimators for the entropy, the Kullback-Leibler divergence (KLD) as well as the mutual information is to apply a nearest neighbor (NN) distance approximation procedure. In what follows, we first introduce this approximation that can be applied for probability density functions (pdf)s. After that, we introduce the estimators and provide additional background information.

3.1. Estimation via NN distances

3.1.1. Notation

Let $X = \{X_1, X_2, \ldots, X_n\}$ with $n \in \mathbb{N}$ be an iid sample with $X_i \sim P$ and let $\|\cdot\|$ be a norm on $\mathbb{R}^d$. Now, $nn_{i,1} \in X$ is the first NN of X_i in that sample if and only if

$$r_{i,1} := \|X_i - nn_{i,1}\| \leq \|X_i - X_j\| \ \ \forall X_j \in X \setminus \{X_i\},$$

with distance $r_{i,1}$. For every positive integer $k < n$, the kth NN $nn_{i,k}$ of X_i is defined equivalently. If we assume $\|\cdot\| = \|\cdot\|_2$, the d-dimensional unit ball has the volume $c_{1,d} := \pi^{d/2}(\Gamma(d/2+1))^{-1}$,where Γ is the Gamma function. The NN distance method takes advantage of the following approximation from Lebesgue's differentiation theorem

$$p(x) \approx \frac{1}{r^d c_{1,d}} \int_{B_r(x)} p(y)dy, \tag{1}$$

where $B_r(x)$ is a ball with a small radius r around x which has the volume $r^d c_{1,d}$. Obviously, equality holds for constant $p(y)$ in $B_r(x)$. The pdf estimator in the pioneering work of Kozachenko and Leonenko (1987) is defined as

$$\hat{p}(X_i) := \frac{1}{(n-1)r_{i,1}^d c_{1,d}}.$$

An intuition is as follows: Let $X = \{X_1, X_2, \ldots, X_n\}$ be as defined above with $X_i \sim P$ and pdf p. Further, let $B_{r_{i,1}}$ be the neighborhood d-ball around X_i. Then, let $X^* \sim P$ be a random variable. The probability p^* for $X^* \in B_{r_{i,1}}$ is $\int_{B_{r_{i,1}}} p(y)dy$. Now, if we were to estimate p^* given the sample $X \setminus \{X_i\}$, we find that 1 out of $n-1$ realizations is inside the closed ball $B_{r_{i,1}}$. Hence, the maximum likelihood estimate (MLE) for p^* is $\frac{1}{n-1}$ and therefore

$$\int_{B_{r_{i,1}}} p(y)dy \approx \frac{1}{n-1}.$$

The rest follows from (1).

3.1.2. Entropy estimation

The entropy estimator of Kozachenko and Leonenko (1987) is derived by averaging over the natural logarithm

$$\hat{H}_n(X) := \frac{1}{n}\sum_{i=1}^{n} \log\left(r_{i,1}^d (n-1) c_{1,d} e^{\gamma}\right), \tag{2}$$

where $\gamma \approx 0.57721$ is the Euler-Mascheroni constant. Alternatively, especially in information theoretic context, $\log_2$ is applied.

The method gained a lot of attention in the statistics community, as it is simple and has favorable statistical properties: It is asymptotically unbiased with vanishing

variance for bounded p and $\exists\epsilon > 0$ such that $E(p^{-\epsilon}) < \infty$ (Leonenko *et al.*, 2006). The latter work also extends (2) to an arbitrary $k < n$, which then suffers from increased bias but has less variance in the scenario of a finite sample size. This is intuitively clear, as the local constancy assumption for $p(x)$ in the neighborhood-ball is violated to a higher degree for a growing distance $r_{i,k}$. However, the robustness of $r_{i,k}$ increases, given more data points $\{nn_1, nn_2, \ldots, nn_k\}$.

3.1.3. MI estimation

For $d \geq 2$, write $X_i = (X_{i,1}, X_{i,2})$ and let X^1 and X^2 be the marginal samples of $\{X_{i,1}\}_{i=1,\ldots,n}$, $\{X_{i,2}\}_{i=1,\ldots,n}$, respectively. A straight forward MI estimator is $\hat{H}_n(X^1) + \hat{H}_n(X^2) - \hat{H}_n(X)$. Instead, the famous work of Kraskov *et al.* (KSG; 2004) uses the flexibility in the choice of k to construct a superior MI estimator when compared to the straight forward approach. The algorithm of KSG is as follows: let k be fixed. Then, a variation of $\hat{H}_n(X)$ is computed w.r.t. the max norm, that is $r^{\infty}_{i,k} = \|X_i - nn_{i,k}\|_{\infty}$. Now, for each $X_{i,1}$, the number of neighbors $k_{i,1}$ is selected as follows: Let $B_{r^{\infty}_{i,k}}(X_i)$ be the max norm ball around X_i. Furthermore, let $B_{r^{\infty}_{i,k}}(X_i)|_{\mathcal{S}^1}$ be the canonical projection of $B_{r^{\infty}_{i,k}}(X_i)$ onto the X^1 subspace. Then, $k_{i,1}$ is the number of elements of X^1 that are in the projected ball. $k_{i,2}$ is defined equivalently (see Kraskov *et al.*, 2004, for an illustrative example of this procedure). This leads to the KSG estimator

$$\hat{I}_{n,k}(X^1, X^2) := \psi(n) - \psi(k) - \frac{1}{n}\sum_{i=i}^{n}\left(\psi(k^*_{i,1}) + \psi(k^*_{i,2})\right),$$

where ψ is the digamma function and $k^*_{i,1} = k_{i,1} + 1, k^*_{i,2} = k_{i,2} + 1$. Note that due to the clever choice of $k_{i,1}$ and $k_{i,2}$, explicit distances cancel out in the formula. KSG provides excellent results if marginal distributions P_1 and P_2 are of low dimension with rather moderate componentwise dependencies.

Amelioration of the NN distance method

Many extensions of KSG exist. Berrett *et al.* (2019) propose a weighted average of pdf estimates derived from $k = 1, 2, \ldots m$ neighbors, with m being small with respect to n. Gao *et al.* (2015); Lord *et al.* (2018) apply a local principle component analysis (PCA) or single value decomposition (SVD) on the kth NNs of X_i. By that, the neighborhood-ball around X_i is replaced with a more suitable structure that captures local dependencies of the data. For example, an ellipsoid in which $p(x)$ is rather constant. At this end, Lu and Peltonen (2020) enhance the PCA-approach by adding a bootstrap procedure to test for potential overcorrection. However, a substitution of the neighborhood-ball comes at the cost of an asymptotical bias.

3.1.4. KLD estimation

A KLD estimator takes two iid samples X with $X_i \sim P$ and Y with $Y_i \sim Q$ into account. For the sake of simplicity, we assume that X and Y are of identical sample size n. The pdf approximation of (1) is not exclusive for $p(x)$. It can also be applied to approximate $q(x)$, although only data points from the X sample are taken into account. Now, a straight forward KLD estimator is

$$\begin{aligned}\hat{D}^W_{n,k}(X,Y) &:= \frac{1}{n}\sum_{i=1}^{n}\log\left(\frac{(r^x_{i,k})^d(n-1)c_{1,d}e^{\gamma}}{(r^y_{i,k})^d n c_{1,d}e^{\gamma}}\right) \\ &= \frac{d}{n}\sum_{i=1}^{n}\log\left(\frac{r^x_{i,k}}{r^y_{i,k}}\right) + \log\left(\frac{n-1}{n}\right),\end{aligned} \tag{3}$$

where $r^x_{i,k}$ and $r^y_{i,k}$ are the distances from X_i to its kth nearest neighbor in X or Y, respectively. This estimator was explicitly proposed by Wang *et al.* (2009). However, its mean square consistency was already provided in the little noticed work of Leonenko *et al.* (2006). More recently, Zhao and Lai (2020) provided convergence rates for bias and variance if pdfs $p(x)$ and $q(x)$ meet some criteria. The authors further showed that the estimator's convergence rate is close to the minimax rate. The minimax rate is, heuristically spoken, the best theoretical performance of an estimator, given the worst case scenario. It is worth noting that (3) explicitly applies NN distances. Therefore, problems of well-definedness appear for data with duplicates as $\exists X_i \in X$ with $r^x_{i,1} = 0$. To prevent $\log(0)$ in (3), we introduced a case discrimination $\max\left\{\frac{r^x_{i,k}}{r^y_{i,k}}, \epsilon\right\}$ in our implementation.

3.2. Derivation of the BannMI empirical hyperparameters

In the case study of our work, we apply BannMI with empirically derived hyperparameters $\{\hat{\alpha}, \hat{\beta}\}$ for the Beta prior-distribution. In what follows we describe this empirical derivation. The approach was suggested by Gelman *et al.* (2015). It uses the method of moments as follows:

1. $\forall X_i \in X$, compute estimates for θ_i via Maximum Likelihood Estimation (MLE). That is

$$\hat{\theta}_{i,\text{MLE}} = \frac{N_{i,k}}{k}.$$

2. Interpret the iid sample with unknown parameters

$$\{\hat{\theta}_{i,\text{MLE}}\}_{i=1,\ldots n} \text{ with } \theta_{i,\text{MLE}} \sim \text{Beta}(\alpha, \beta).$$

3. Estimate mean and variance of the sample

$$\hat{\mu} = \frac{1}{n}\sum_{i}^{n}\hat{\theta}_{i,\text{MLE}} \quad \text{and} \quad \hat{\sigma}^2 = \frac{1}{n}\sum_{i}^{n}(\hat{\theta}_{i,\text{MLE}} - \hat{\mu})^2.$$

4. Apply a transformation to receive estimates for the parameters

$$\hat{\alpha} = \frac{\hat{\beta}\hat{\mu}}{1-\hat{\mu}} \quad \text{and} \quad \hat{\beta} = \frac{1}{\hat{\sigma}^2}\left(\hat{\mu} - \hat{\mu}^2 - \hat{\sigma}^2\right)(1-\hat{\mu}).$$

By that, $\{\hat{\alpha}, \hat{\beta}\}$ are plugged into the BannMI.

4. Supplementary material on the benchmark study

In the Method parts of our work we derived two KLD estimators: A frequentist NN ratio based KLD estimator that was inspired by the f-divergence estimator presented by Noshad *et al.* (2017) and our BannMI. For simplicity, we refer to the estimators NMI and BannMI – which is identical to their MI application, although KLD is estimated. Next, we first benchmark the estimators to derive an optimal parameter setting (Section 3.1.4). With the optimal estimator parameters at hand, we proceed to benchmark for MI in our main work. Furthermore, Section 4.2 provides detailed performance information about the MI benchmark with Gaussians.

param.	BannMI	NMI
d	$2, 5, 10$	$2, 5, 10$
k	$5, 10, 20, 50$	$10, 20, 50, 150$
ϵ	–	eps, adapt, 10^{-6}, 0.01
$(\hat{\alpha}, \hat{\beta})$	0.1, 0.2, 0.5, 1, emp.	–

Table 1. For each estimator, all combinations of parameters were tested. For the non-empirical hyperparameter setting in BannMI, it holds that $\hat{\alpha} = \hat{\beta}$. For NMI in the ϵ-scenario, "eps" is the machine epsilon and adapt$=\frac{1}{nd}$, where n, d are sample size and data dimensionality. The slight difference in k for both methods is due to empirical testing: We wanted to make sure, that the minimum for each estimator with frozen parameters despite the parameter k, is not at the edge of our testing range. For example: $k = 5$ does not minimize the mean squared error for BannMI, neither does $k = 50$.

4.1. KLD benchmark on pairs of non-negative random variables

4.1.1. Data generating proess (DGP):

We simulate componentwise uncorrelated random variables (RV)s with dimension $d \in \{2, 5, 10\}$ of the Exponential, Lognormal, Rayleigh, Gamma and Chi-squared distributions with various parameter settings. The samples are grouped into all feasible tuples (drawing with replacement). For example, $(exp(1), exp(2))$ refers to a tuple of two exponentially distributed samples with parameters $\lambda_1 = 1, \lambda_2 = 2$ and so on. We then apply the KLD estimators with all possible combinations of parameter settings as shown in Table 1. The sample size for all scenarios is $n = 1,000$. A detailed evaluation for both KLD estimators is shown for $d = 2$ with number of neighbors $k = 50$, see Figure 3 and the Tables 2 and 3.

4.1.2. Main results:

All hyperparameter settings for BannMI approximately halve the mean squared errors compared to NMI with its respective parameter settings, see Tables 2 and 3. These tables also demonstrate that the 0-scenarios (two identical distributions) are comparable for both estimators. Further, other parameter scenarios produce comparable results.

4.2. Key performance indicators of MI benchmark in Gaussian setting

In the Gaussian DGP of the Benchmark part of our work, we tested estimators (u)BannMI, KSG, WMI, NMI with optimized parameter settings derived from Section 3.1.4 on data of dimension $d = \{2, 5, 10\}$. As for BannMI, the empirircal parameter setting performed comparable to an uninformative prior with $\hat{\alpha} = \hat{\beta} = 0.1$, we included both scenarios. Results of the key performance indicators mean squared error (MSE), standard derivation and Pearson correlation are shown in Figure 4. The estimator WMI had to be excluded for the Pearson correlation due to its production of NaN values and numerical instabilities.

5. Supplementary material on the case study

To further investigate the combinatorial effect of the EfAs on the programmed cell death, we trained random forests to predict apoptosis on healthy cells via the Python package `RandomForestClassifier`.

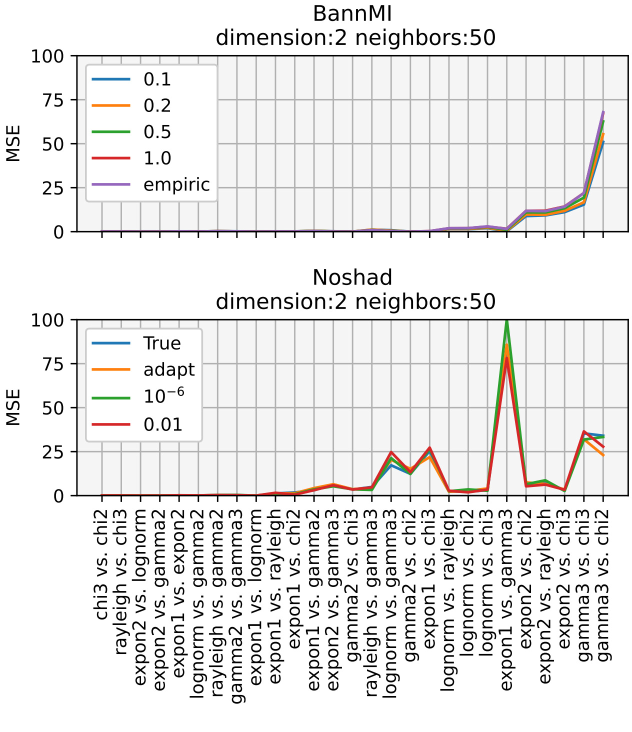

Fig. 3. Performance example with sample size $n = 1,000$ and amount of neighbors considered $k = 50$. Per distributional tuple, the mean squared error between 25 estimates and the numerical result (`nquad`) was derived. X-axis depicts KLDs based on non-identical distributions, results are sorted with respect of increasing KLD value, which means that they were sorted based on the numerical result of the tuple. Other parameter sample sizes and other values for k produce comparable results.

$(\hat{\alpha}, \hat{\beta})$	0.1	0.5	1	*emp.*
diverse	3.873511	4.724244	5.127471	5.144825
identical	0.001694	0.001743	0.001589	0.001682

Table 2. Average mean squared errors derived from BannMI estimates compared to estimates from numerical integration. Due to the componentwise independence of the distributions applied, the numerical integration could be reduced to an 1D scenario with neglectible standard errors. The quantity to estimate is KLD between sampled tuples of pairwise different non-negative distributions (diverse) and sampled tuples of a non-negative distribution (identical). The average mean squared errors were computed with respect to all tuples, whereas each tuple was drawn 25 times. Here, the column for the parameter 0.2 is not shown for brevity. The further parameters of this computation were as follows: sample size: 1,000, sample dimensionality: 2, nearest neighbors: 50. Other further parameter settings produce comparable results (not shown).

5.1. Random forests application

We selected time point $t = 9$, as most apoptotic cells are found in that joint sample of the control cell lines. We defined apoptosis as a cleaved Caspase-3 level above 5 and used 40% of the data for training. To avoid overfitting, we restricted the tree depth to four. For each 4D EfA combination, the following four random forests were trained: 1, default parameter setting. 2, entropy as split quality measure. 3, entropy as split quality measure and balanced class weight. 4, entropy as split quality measure and application balanced subsamples.

ϵ	eps	adapt	10^{-6}	0.1
diverse	9.467923	9.158444	10.029276	9.303394
identical	0.001760	0.001662	0.001836	0.001668

Table 3. Average mean squared errors derived from NMI estimates compared to estimates from numerical integration. For further information, see description of 2.

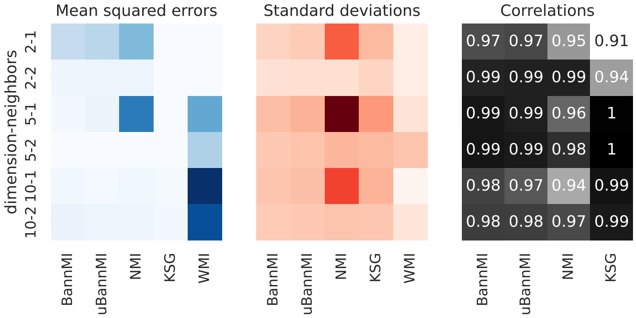

Fig. 4. For each setting with the 62 covariance matrices, we computed 25 estimates and derived the mean. Here, the MSE of each cell is with respect to the 62 mean estimates per dimension and neighbor index. In the standard deviations benchmark, each cell is a mean of the 62 standard deviations that were derived from the 25 repeated experiments. In the Pearson correlation table, a cell represents the correlation between the 62 true values/mean estimates.

AUC-best (second best) model	true apop.	true non-apop.
pred. apop.	558 (223)	5,076 (1)
pred. non-apop	51 (386)	48,905 (53,980)

Table 4. Confusion matrices: For the AUC-best model, the unequal class weight of apoptotic and non-apoptotic cells was balanced (model 3, AUC=0.9679), which led to a slightly better performance than model 2 (unbalanced class weight, AUC=0.9674).

The best and second best performance is received for the feature combination of phosphorylated ERK, p38, AMPK, STAT1 with AUC as benchmark criterion, see the confusion matrix in Table 4. Here, we want to raise attention to the sensitivity of our second best model, which is > 0.999. Thus, these particular combinations of protein phosphorylation in healthy cells are found exclusively in their apoptotic subset. A multivariate logistic regression for all 4D EfA combinations performed poorly (data not shown). After ranking the models with respect to AUC, we computed the average feature importance per EfA of the 25 top performing models, see Figure 5.

5.2. KLD for differential expression

Differential expression (DE) is relevant in single cell analysis with yet no clear state-of-the-art method for its quantification (Hie *et al.*, 2020). However, the KLD is a measure for dissimilarity between distributions and therefore well suited to analyse multivariate DE between phosphoproteomic samples. Next, although not in the context of a testing procedure (with a p-value as result), we use BannMI as KLD estimator to quantify DE between the control phenotypes in a 4D setting.

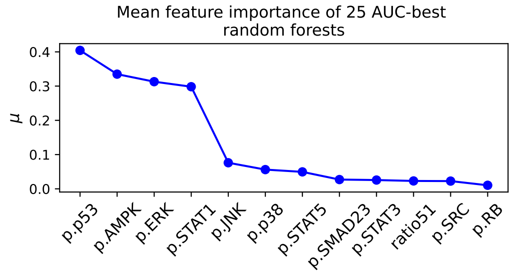

Fig. 5. The phosphorylated proteins p53, AMPK, ERK and STAT1 have the largest effect on the prediction outcome in a 4D setting.

To do so, we computed KLD between the apoptotic cells and the proliferating cells, as well as between the apoptotic cells and the resting cells. Again, we ranked results with respect to KLD and selected the 25 highest scores to count the individual EfAs in the occurring quadruples. Results are shown in Figure 6 (left). Intriguingly, the four most frequently occurring EfAs, which are AMPK, p53, STAT1 and ERK in the "apoptotic vs. resting" scenario, are identical with the phosphoproteins of the highest feature importance as shown in Figure 5.

In our final analysis, we were interested in the 4D DE when comparing the cancerous to the control cells per phenotype, see Figure 6 (right). To do so, again our EfA occurrence ranking procedure was applied. Besides a strong DE of the metabolic pathway (AMPK) and of the cell stress pathway (p53), our KLD estimations show a robust difference of GSK3β phosphoylation in cancer when compared to the control. As stated in our main work, the protein plays a role in DNA repair. Here, GSK3β expression refers to its phosphorylation at Ser9, a phosphorylation site that inactivates the kinase. Compared to control cells, in cancer cells GSK3β Ser9 expression is approximately twice as high (data not shown). However, it is thinkable that overall GSK3β expression is leveraged in cancer, due to a high DNA damage frequency.

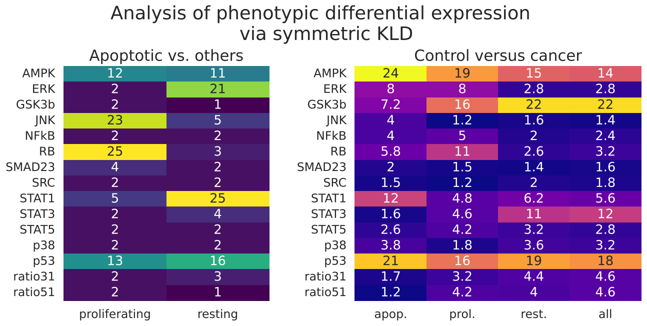

Fig. 6. EfA occurrence DE rankings with respect to cell phenotypes. Left: DE between apoptotic and proliferating/resting control cells. Right: DE between control and cancer. To do so, 4D DE between control and every cancer subtype was derived and an EfA occurrence ranking was performed. Then, the five EfA occurrence rankings were averaged for each phenotype.

References

Berrett, T. B. *et al.* (2019). Efficient multivariate entropy estimation via k-nearest neighbour distances. *The Annals of Statistics*, **47**(1), 288–318.

Gao, S. *et al.* (2015). Efficient estimation of mutual information for strongly dependent variables. In *Artificial Intelligence and Statistics*, pages 277–286. PMLR.

Gelman, A. *et al.* (2015). *Bayesian Data Analysis*. CRC Press, New York, 3 edition.

Hie, B. *et al.* (2020). Computational methods for single-cell rna sequencing. *Annual Review of Biomedical Data Science*, **3**, 339–364.

Kozachenko, L. and Leonenko, N. (1987). Sample Estimate of the Entropy of a Random Vector. *Problems of Information Transmission*, **23**(2), 95–101.

Kraskov, A. *et al.* (2004). Estimating mutual information. *Physical Review E*, **69**, 066138.

Leonenko, N. *et al.* (2006). Estimation of entropies and divergences via nearest neighbors. In *ProbaStat 2006*, volume 39, pages 265–273.

Lord, W. M. *et al.* (2018). Geometric k-nearest neighbor estimation of entropy and mutual information. *Chaos: An Interdisciplinary Journal of Nonlinear Science*, **28**(3), 033114.

Lu, C. and Peltonen, J. (2020). Enhancing nearest neighbor based entropy estimator for high dimensional distributions via bootstrapping local ellipsoid. In *Proceedings of the AAAI Conference on Artificial Intelligence*, volume 34, pages 5013–5020.

Noshad, M. *et al.* (2017). Direct estimation of information divergence using nearest neighbor ratios. In *2017 IEEE International Symposium on Information Theory (ISIT 2017)"*, pages 903 – 907, Aachen, Germany. Institute of Electrical and Electronics Engineers (IEEE).

Wang, Q. *et al.* (2009). Divergence Estimation for Multidimensional Densities Via k-Nearest-Neighbor Distances. *IEEE Transactions of Information Theory*, **55**(5), 2392–2405.

Zhao, P. and Lai, L. (2020). Minimax optimal estimation of kl divergence for continuous distributions. *IEEE Transactions on Information Theory*, **66**(12), 7787–7811.
